# Non-Isothermal Crystallization Kinetics and Properties of CaO-Al_2_O_3_-SiO_2_ (CAS) Glass-Ceramics from Eggshell Waste, Zeolite, and Pumice

**DOI:** 10.3390/ma17225630

**Published:** 2024-11-18

**Authors:** Bahadır Aydın, Hüseyin Özkan Toplan, Nil Toplan

**Affiliations:** 1 Metallurgy and Materials Engineering Department, Engineering Faculty, Esentepe Campus, Sakarya University, 54187 Sakarya, Türkiye; toplano@sakarya.edu.tr (H.Ö.T.); toplan@sakarya.edu.tr (N.T.); 2 Department of Engineering Science, Faculty of Engineering, İstanbul University-Cerrahpaşa, 34320 İstanbul, Türkiye

**Keywords:** crystallization kinetics, CAS glass-ceramics, mechanical properties, physical properties

## Abstract

In this study, the crystallization behavior, microstructure, and mechanical and physical properties of CaO-Al_2_O_3_-SiO_2_ (CAS)-based glass-ceramics prepared from eggshell waste, zeolite, and pumice were investigated using X-ray diffraction (XRD), differential thermal analysis (DTA), scanning electron microscopy (SEM), a nanoindentation tester, and the Archimedes method. XRD analysis revealed that anorthite and wollastonite crystalline phases precipitated in the glass-ceramic samples after sintering at temperatures of 1000 °C and 1100 °C. However, diffraction peaks belonging to the wollastonite phase disappeared after sintering at 1200 °C, while peaks representing the pseudowollastonite phase were detected together with anorthite in the samples. SEM images showed that the crystals become coarser as the sintering temperature increased, with the crystal morphology transitioning from needle-like to rod-like. The crystallization activation energy (*E*_a_) and Avrami parameter (*n*), both kinetic parameters, were calculated from DTA curves plotted at different heating rates using the Kissinger, Ozawa, and Matusita approaches. The results indicated that the crystallization activation energy of the CASZ glass ranged from 406 to 428 kJ mol^−1^, while that of the CASP glass varied from 356 to 378 kJ mol^−1^, depending on the method used. Additionally, the Avrami constant (*n*) was calculated to be 3.33 for CASZ and 2.89 for CASP. The hardness and bulk density of the glass-ceramic samples were significantly affected by the porosity present in the structure, with the highest hardness and bulk density values achieved for the CASZ glass-ceramic sample at the initial sintering temperature of 1000 °C.

## 1. Introduction

Glass-ceramics are materials fabricated through the controlled nucleation and subsequent crystallization of suitable glass compositions and contain one or more crystalline phases [1]. However, it is well known that glass-ceramics are not entirely crystalline materials, usually 50–95 vol% crystalline with the remainder being a residual glassy matrix [2]. The properties of synthesized glass-ceramics vary depending on the base glass chemical composition, precipitated crystalline phases, the amount of residual glass, and the distribution of crystals in the microstructure [3]. Numerous glass-ceramic systems including MgO-Al_2_O_3_-SiO_2_ (MAS), Li_2_O-Al_2_O_3_-SiO_2_ (LAS), BaO-Al_2_O_3_-SiO_2_ (BAS), and CaO-Al_2_O_3_-SiO_2_ (CAS) have been developed for both academic research and commercial applications [4]. Among them, CaO-Al_2_O_3_-SiO_2_ (CAS)-based glass-ceramics have found a wide variety of applications due to their high mechanical properties, low thermal expansion coefficient (CTE), shining appearance, and good chemical resistance [5,6]. These features make it possible to use these materials as decorative materials, floor tiles, claddings, etc., for the industry [6,7]. CAS system glasses can be converted into glass-ceramics when subjected to appropriate heat treatment conditions [5]. It has been mentioned in the literature that some oxides like TiO_2_, CaF_2_, ZrO_2_, P_2_O_5_, Cr_2_O_3_, Fe_2_O_3_, B_2_O_3_, ZnO, and Li_2_O can be utilized as nucleating agents to facilitate crystallization and lower the crystallization temperature of CAS glasses [4,8].

Differential thermal analysis (DTA) is an effective and practical method for examining the crystallization kinetics of glasses. Generally, both isothermal and non-isothermal techniques can be employed to study the crystallization kinetics of glasses, while the non-isothermal method which uses a constant heating rate is a more commonly used technique due to it giving flexibility and simplicity for determining cooling and heating rates. The Avrami parameter (*n*) and crystallization activation energy (*E*_a_) are key kinetic parameters to evaluate the crystal growth mechanism and crystallization ability of glasses. These parameters are calculated using appropriate equations with the data obtained from DTA results at different heating rates [9,10,11,12].

The utilization of numerous domestic and metallurgical wastes, such as incinerator fly ash, rice husk ashes, blast furnace slag, coal fly ash, steel fly ash, etc., have been reported for CAS glass-ceramic production as raw materials due to contain SiO_2_, Al_2_O_3,_ and CaO oxides in a high quantity [13]. Eggshells are classified as agricultural waste and discarded annually worldwide in large quantities of about 250,000 tons by the food industry [14,15]. Chicken eggshells contain about ~94 wt.% CaCO_3_, 1 wt.% tri-calcium phosphate, 1 wt.% MgCO_3,_ and about 4 wt.% proteins and hold huge potential as an alternative source of CaO for CAS glass-ceramics [16].

Different natural igneous rocks (e.g., granite, basalt, syenite, etc.) can be used as raw materials in the production of glass-ceramics because of their high silica content. In this context, natural zeolite clinoptilolite, which is formed from the interactions of volcanic rocks and ash with water, is a candidate material with high silica and alumina content. Besides the silica and alumina contents, natural zeolites comprise other oxides such as Fe_2_O_3_, K_2_O, CaO, MgO, and Na_2_O in the amount of 1–5 wt.% [17]. Similarly, pumice, which originates from volcanic eruptions, is a lightweight natural material with a sponge-like nature and is primarily composed of silica and alumina (wt.% of 60–75% SiO_2_, 13–17% Al_2_O_3_), along with lower contents of Fe_2_O_3_, CaO, Na_2_O, K_2_O, and TiO_2_. Therefore, it offers promising potential as a raw material and is utilized in various fields, including the construction, cosmetics, dental, ceramic, and glass industries, due to its affordability and accessibility [18,19,20].

The literature discusses the use of eggshells as an alternative low-cost source of CaO in various studies, including those on glass-ceramic orbital implants [21], bioactive glass-ceramic systems [22,23], hydroxyapatite artificial bone [21], and dielectric material production [24]. Specifically, regarding the CAS system, studies such as that of Hossain et al. [16] have investigated the properties of pseudowollastonite and wollastonite-based glass-ceramics derived from solid waste, and the work of Hongxu et al. [25] examined the impact of the sintering temperature on the properties of wollastonite-based glass-ceramics produced from waste materials. Furthermore, the more widespread investigation of this agricultural waste focuses particularly on its application in the production of glass-ceramic foams using foaming agents. Research on the use of pumice and zeolite in the preparation of glass and glass-ceramic compositions remains sparse in the literature. Studies have documented the synthesis of glass-ceramic foams from natural pumice, the incorporation of fluxing agents in wall tile glazes, and the feasibility of utilizing pumice in the glaze composition of vitrified products [26,27,28]. Similarly, several investigations have centered on zeolite concerning the production of foam glass-ceramics [29,30,31] and the treatment of radioactive waste [32,33].

To our knowledge, the production of high-value materials such as glass-ceramics using entirely waste and natural raw materials is limited in existing research. This study utilizes eggshell waste, along with zeolite and pumice, as substitutes for pure chemical reagents in the preparation of value-added CAS-based glass-ceramics. The effect of these natural raw materials on the crystallization behavior and properties of the glass-ceramics was investigated by substituting zeolite with pumice in the selected CAS composition. Notably, the synthesized glass-ceramics are cost-effective compared to conventional or scientific-purpose glass-ceramics that utilize pure oxides, as the natural raw materials employed are abundant and affordable volcanic rocks found in Türkiye.

## 2. Materials and Methods

**Figure 1:**
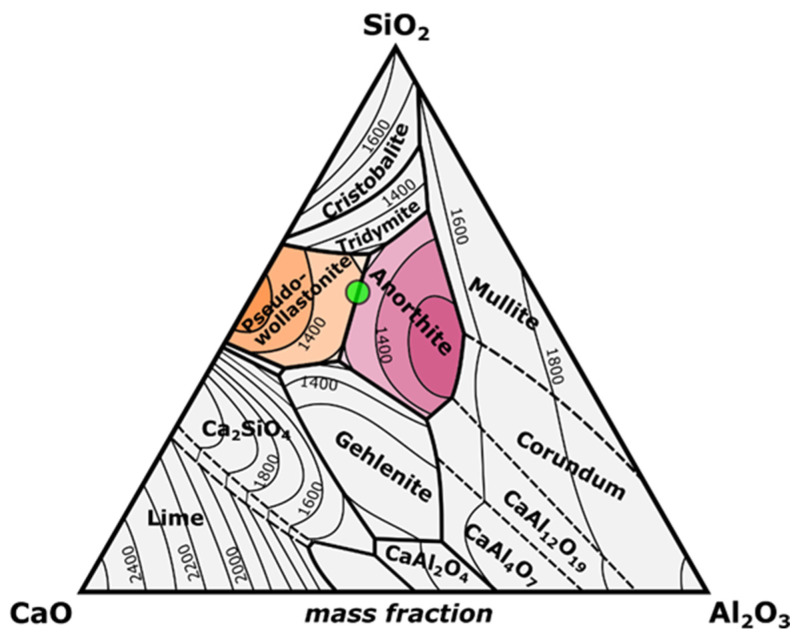
The ternary phase diagram of the CaO-Al_2_O_3_-SiO_2_ system and the experimental CAS composition (shown as a green circle) on the wollastonite–anorthite phase boundary.

Two experimental CaO-Al_2_O_3_-SiO_2_ (CAS) batches were prepared using eggshell powder and natural zeolite and pumice raw materials according to the base composition listed in Table 1. Chicken eggshell powder is incorporated in both CASZ- and CASP-coded batches as a source of CaO, while zeolite (Gördes/Manisa province, Türkiye) and pumice (Nevşehir province, Türkiye) volcanic rocks were utilized as sources of alumina and silica in CASZ and CASP batches, respectively. Additionally, aluminum oxide powder with ≥90% purity (Merck (Rahway, NJ, USA), CAS No. 1344-28-1) was added externally to the batches to compensate for the deficient amount of alumina that could not be derived from zeolite and pumice. The reasonable explanation for the selection of this composition in the present study is based on the fact that it is a low-temperature anorthite (CaAl_2_Si_2_O_8_) + wollastonite (CaSiO_3_) formation region according to the CAS ternary diagram (Figure 1).

Raw materials were precisely weighed according to the selected CAS composition given in Table 2 and then homogenized by ball milling at a speed of 250 rpm for 24 h. Approximately 100 g batches were placed in alumina crucibles and initially heated in an electric furnace from room temperature to 900 °C at a heating rate of 10 °C min^−1^. At this temperature, the mixtures were calcined for 1 h to allow for the decomposition of carbonates. Following this, the temperature was continuously increased to 1450 °C, and melting was carried out for 2 h under atmospheric conditions. Subsequently, to prevent any crystallization, the melts were rapidly quenched in distilled water at room temperature, resulting in the formation of glass frits. The obtained glass frits were ground in a planetary ball mill (Fritsch Pulverisette 6, Idar-Oberstein, Germany) at 600 rpm for 15 min, and then sieved to achieve a particle size of less than 45 μm. After that, CAS-coded glass powders were shaped with a uniaxial hydraulic press under a load of 115 MPa to form glass compacts with a diameter of 10 mm.

Differential thermal analysis (DTA, Netzsch STA 449, Waldkraiburg, Germany) was performed to determine the glass transition (T_g_) and crystallization (T_c_) temperatures of glass samples and for kinetic studies. For this purpose, CAS-coded glass powders were heated from room temperature to 1250 °C with a heating rate of 5, 10, 15, and 20 °C min^−1^ in the air, using alumina powder as the reference material.

**Figure 2:**
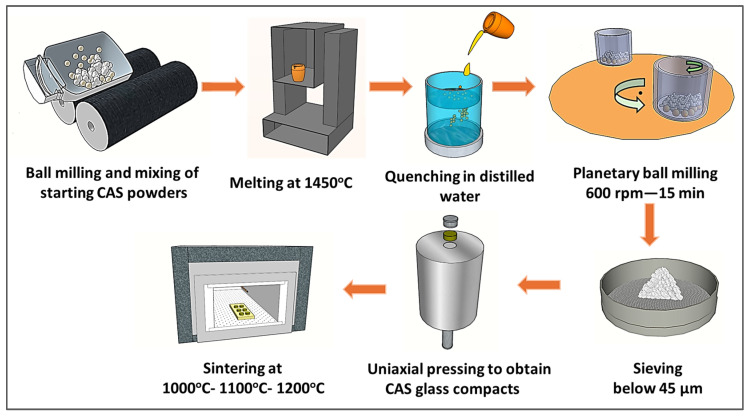
Schematic representation of the production process of CAS glass-ceramics.

Glass-ceramics were produced by sintering the disk-shaped CAS glass compacts at temperatures ranging from 1000 to 1200 °C, with a heating rate of 10 °C min^−1^ for 1 h, taking into account the T_c_ temperatures determined from the DTA results. Figure 2 briefly shows the fabrication process of CAS-coded glass-ceramics.

An X-ray diffractometer (XRD, RIGAKU D/Max/2200/PC, Tokyo, Japan) was utilized to determine crystalline phases that occurred in the produced glass-ceramics. The XRD analysis was performed on glass-ceramics compacts in the range of 10–80° with Cu K_α_ radiation (λ = 1.54056 nm) at a scanning rate of 2°/min. Energy-dispersive X-ray spectroscopy (EDS)-equipped scanning electron microscopy (SEM, Jeol 6060LV, Tokyo, Japan) was used to examine the glass-ceramic morphology, crystal sizes, and their distribution. Microstructure examinations were conducted on the polished surfaces of the glass-ceramics. The surfaces of the specimens were coated with a thin gold layer using the sputtering method to eliminate the charging effect before SEM investigations. Fourier transform infrared spectroscopy (FTIR, PerkinElmer Spectrum Two, Waltham, Massachusetts, USA) was performed in the range of 1400–400 cm^−1^ to investigate the effect of the used natural raw materials on the structural properties of the starting glasses.

The mechanical behavior of CAS glass-ceramics was evaluated using a nanoindentation tester (Anton Paar NHT^3^, Graz, Austria) with a Berkovich diamond tip. Nanoindentation tests were performed with a 10 s holding time and a maximum load of 50 mN, at a loading/unloading rate of 100 mN/min, assuming the material’s Poisson ratio of 0.245 [34].

The Archimedes method was used to calculate the bulk density (*ρ*) and apparent porosity (*φ_a_*) of the glass-ceramics specimens. The dry weights (*w_d_*) were measured using an analytical balance after drying the sintered samples in an oven at 100 °C for 24 h. Afterwards, the samples were immersed in distilled water at room temperature for 24 h to obtain their immersed weights (*w_i_*) and wet weights (*w_w_*). The apparent porosity and bulk density were calculated from the experimental data using the following equations:

(1) $$\rho =\frac{w_{d}}{w_{d}-w_{i}}\times \rho_{water}\;\;\;\;$$

(2) $$\varphi_{a}=\frac{w_{w}-w_{d}}{w_{w}-w_{i}}\times 100\;\;\;\;\;\;\;\;\;$$

## 3. Results and Discussion

### 3.1. X-Ray Diffraction Analysis of CAS Glasses and Glass-Ceramics

**Figure 3:**
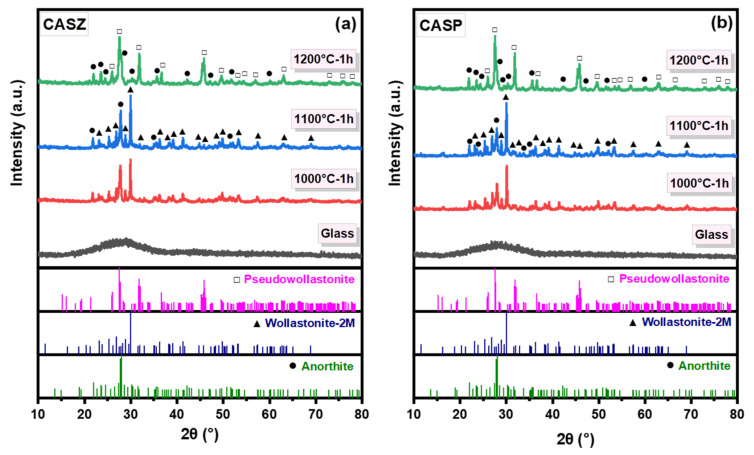
XRD patterns of (**a**) CASZ and (**b**) CASP glass and glass-ceramic samples sintered at different temperatures for 1 h.

Figure 3 shows the X-ray diffraction (XRD) patterns of the CASZ and CASP glasses, as well as the glass-ceramics, after sintering at temperatures between 1000 and 1200 °C for 1 h. The as-melted samples exhibited a broad hump centered at approximately 2θ = 28°, indicating the completely amorphous nature of the material. At the initial sintering temperature of 1000 °C, the main crystalline phases detected in CASZ and CASP glass-ceramics were wollastonite-2M (CaSiO_3_, ICDD No. 00-027-0088) and anorthite (CaAl_2_Si_2_O_8_, ICDD No. 00-041-1486). Both glass-ceramic compositions generally exhibited similar XRD patterns, with an increase in the intensity of anorthite and wollastonite diffraction peaks as the sintering temperature rose from 1000 to 1100 °C, indicating an improvement in crystallinity. Upon increasing the sintering temperature to 1200 °C, the diffraction peaks corresponding to the wollastonite phase, which were previously observed at various 2θ angles, were no longer detectable. Instead, new diffraction peaks associated with the pseudowollastonite phase (CaSiO_3_, ICDD No. 01-089-6463) emerged at approximately 2θ = 25.6°, 27.6°, 31.8°, 36.7°, 45.8°, 49.6°, 53.4°, 54.4°, 56.9°, 63.09°, 72.90°, 75.9°, and 77.9°. As is known, pseudowollastonite is the high-temperature form of wollastonite, and according to the literature the polymorphic transformation temperature of wollastonite to pseudowollastonite is approximately 1125 °C [35]. Additionally, the number of crystalline peaks representing the anorthite phase increased at this sintering temperature, which can be discerned from the new peaks observable at 2θ = 23.51°, 24.4° 30.3°, 35.57°, 42.2°, 47.1°, and 60.1° angles. This suggests that the high sintering temperature conditions facilitate the growth of anorthite crystals. Glasses in the CAS system have [SiO_4_] and [AlO_4_]Ca[AlO_4_] structural units, and according to the concept of the stable energy of glass structural units, structural changes during crystallization in this system preferably start in [AlO_4_]Ca[AlO_4_], since its stable energy is lower. In this case, free Ca^2+^ ions tend to unite with [SiO_4_] to form wollastonite first. After the precipitation of wollastonite, the [AlO_4_]Ca[AlO_4_] structural units are rearranged and forced to unite with [SiO_4_], leading to the precipitation of anorthite [36].

### 3.2. Microstructural Evaluation of CAS Glass-Ceramics

**Figure 4:**
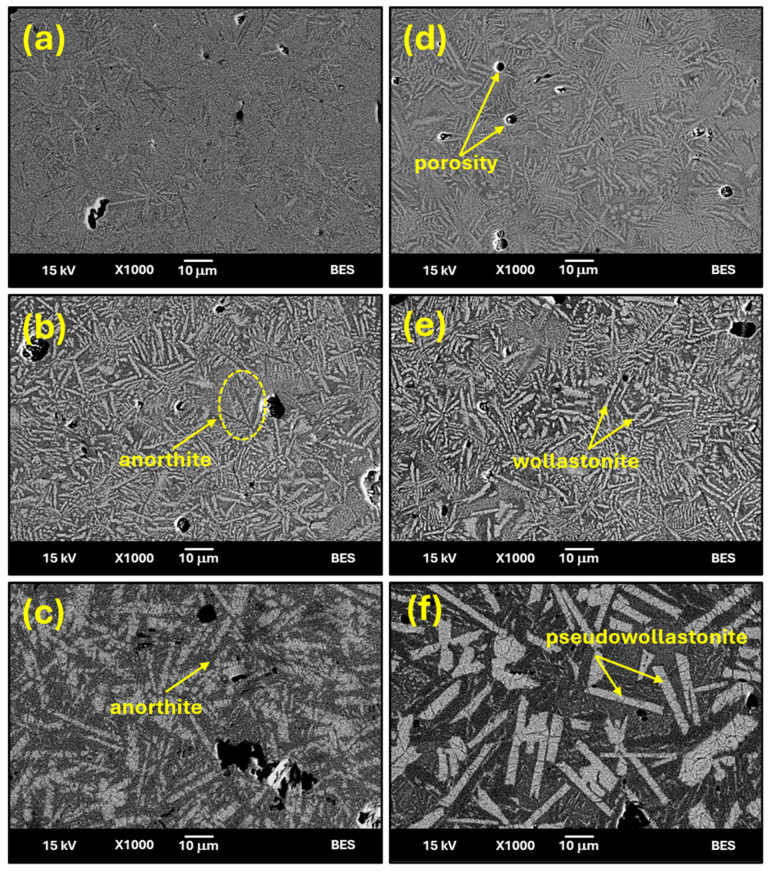
SEM images of the produced CASZ and CASP glass-ceramic samples sintered at different sintering temperatures for 1 h ((**a**). CASZ—1000 °C; (**b**). CASZ—1100 °C; (**c**). CASZ—1200 °C; (**d**). CASP—1000 °C; (**e**). CASP—1100 °C; (**f**). CASP—1200 °C).

Backscattered SEM images of CASZ and CASP glass-ceramic specimens sintered at 1000 °C, 1100 °C, and 1200 °C, as shown in Figure 4a–e, directly compare the effects of the sintering temperature on the microstructure of the glass-ceramics. As can be observed, glass-ceramic samples present porous microstructures, while the porosity content increases as the sintering temperature rises. While this finding appears to contrast with literature reports indicating that the density of glass-ceramics typically increases with higher sintering temperatures, it can be attributed to the rapid decrease in specific volume during the transition from glass to glass-ceramic. As is known from the volume–temperature diagram, the amorphous structure occupies more specific volume than the crystalline structure. When an appropriate parent glass is heat-treated at a certain temperature to be transformed into a glass-ceramic, the increasing temperature primarily causes an increase in specific volume, while crystals that nucleate and grow within the glass after a sufficient time occupy a lower specific volume than the glassy state. Thus, this reduces the content of the glass phase in the structure during the crystallization process, and the viscous flow and densification cannot occur quickly enough [37,38]. This phenomenon will be discussed in detail in the Physical Characterization Section.

SEM images of CAS-coded glass-ceramic samples sintered at 1000 °C for 1 h reveal the precipitation of white needle-like wollastonite and dark-colored needle-like anorthite crystals within the microstructure. However, the number and size of needle-like crystals observed in the CASZ sample at this sintering temperature are relatively small in comparison to CASP. Although not clearly distinguishable, fine-grained white crystals, believed to be predominantly wollastonite, can be identified in the microstructures. This indicates that crystal growth in the CASP composition occurs more readily at the initial sintering temperature of 1000 °C compared to CASZ. In CASP glasses, the higher content of network-modifying oxides resulting from the starting raw material used, compared to CASZ, does not lead to changes in the morphology of the precipitated crystal phases. However, the results indicate that the crystallization tendency of CASP glasses is significantly higher at the same sintering temperature. The reduction in viscosity during the nucleation and crystallization stages in these network modifier-rich glasses facilitates crystal growth [39]. Conversely, in CASZ glasses, where the content of network modifiers is lower, the elevated activation energy required for atomic migration makes crystal growth more challenging. Consequently, this results in the formation of larger crystals in CASP glass-ceramics at the same sintering temperature. Increasing the sintering temperature to 1100 °C resulted in an increase in the size of the anorthite and wollastonite crystals, while their numbers decreased. At elevated sintering temperatures, the reduction in viscosity enhances mass transport, thereby facilitating crystal growth [40]. Following sintering at 1200 °C, the needle-like wollastonite formations were no longer observable and were replaced by large rod-like pseudowollastonite crystals. Additionally, the anorthite crystals, which developed side by side with the pseudowollastonite crystals, had undergone a transformation from needle-like to rod-like structures. Overall, the CASP specimen at this sintering temperature yielded larger crystals.

**Figure 5:**
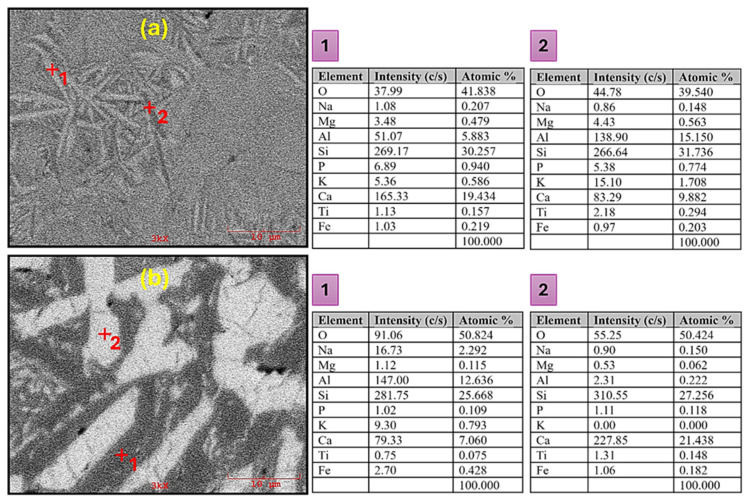
SEM images of the (**a**) CASZ glass-ceramic sample sintered at 1000 °C and (**b**) CASP glass-ceramic sample sintered at 1200 °C, and EDS point analysis of the selected crystals.

In order to better verify the crystal structures determined by XRD and low-magnification SEM images, the elemental composition of the crystals was detected by EDS analyses. Figure 5 shows the EDS analyses of CASZ (1000 °C—1 h) and CASP (1200 °C—1 h) glass-ceramic samples, while the atomic percentage of each element at selected points is given in the table attached to the relevant images. The EDS analysis of the white needle-like crystal (point 1) in Figure 5a reveals high Ca (at. 19.43%) along with Si (at. 30.2%) and O (at. 41.8%) contents, representing wollastonite. The detected values are very close to the theoretical chemical composition of the wollastonite crystal phase with an atomic ratio of Ca:Si = 1:1, and wollastonite crystals with needle-like morphology are widely reported in the literature [41,42]. However, the analysis also revealed the presence of minor quantities of Al (at. 5.8%), which can be ascribed to the dissolution of aluminum as a solid solution in the wollastonite crystals during the sintering process [43]. The dark needle-like crystal (point 2) comprises high concentrations of Ca, Si, Al, and O elements, but has a significantly lower Ca content (9.8 at. %) and a notably higher Al content (15.1 at. %) compared to the analysis of point 1. This finding is in accordance with the morphology and content of anorthite crystals as reported by various researchers [44,45]. In Figure 5b, the dark-colored crystal represents anorthite, while the white crystal with thick rod-like morphology indicates pseudowollastonite with high Ca, Si, and O element content. It is noteworthy that this crystal contains a markedly low Al content, in contrast to that observed in the wollastonite crystal.

### 3.3. Differential Thermal Analysis (DTA)

**Figure 6:**
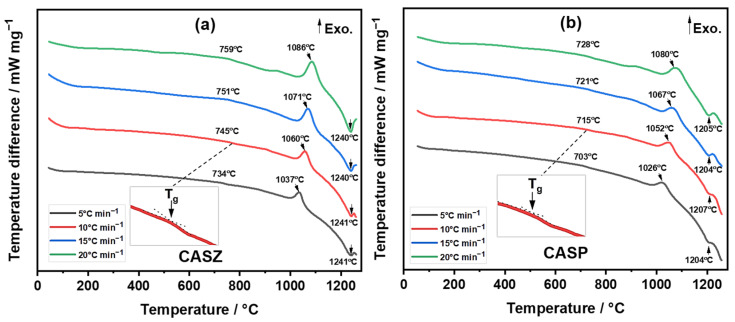
DTA curves of (**a**) CASZ and (**b**) CASP glass powders at different heating rates (5, 10, 15, and 20 °C min^−1^).

Figure 6 presents the DTA results of the CASZ and CASP glass powders at heating rates of 5, 10, 15, and 20 °C min⁻^1^. The curves display two endothermic peaks: the first corresponds to the glass transition temperature (T_g_), while the second indicates the melting temperature (T_m_), which was determined from the summit of the melting endotherm. Additionally, a single exothermic peak signifies the crystallization temperature (T_p_) associated with the formation of anorthite and wollastonite phases. The determined characteristic temperatures of CAS glasses at different heating rates are summarized in Table 3.

As DTA analyses revealed, the glass transition and crystallization peak temperatures for CASZ glass at a 10 °C min^−1^ heating rate were recorded as 745 °C and 1060 °C, respectively, while the aforementioned temperatures were detected as 715 °C and 1052 °C at the same heating rate for CASP glass. Similarly, the decreasing trend in the T_g_ and T_p_ temperatures of CASP glasses was maintained, as compared to those of CASZ glasses at the same heating rates. This behavior can be explained by the fact that the prepared CAS compositions contain some metal oxides which act as network modifiers in different proportions depending on the raw materials used, and the CASP glass comprises more of these modifiers. The introduction of these oxides to the silicate network breaks the strong Si-O-Si covalent bonds and creates non-bridging oxygen (NBO) groups. The existence of NBO groups in the structure weakens the connectivity of the glass network, and the viscosity of the glass decreases, while its tendency to crystallize at lower temperatures increases [46].

### 3.4. Non-Isothermal Crystallization Kinetics

The non-isothermal crystallization kinetics of CAS glasses were studied using the DTA technique at different heating rates. Different approaches were used to estimate the kinetic parameters and to compare the accuracy of the results. The crystallization activation energy (*E*_a_) of CAS glasses was determined using the Kissinger, Ozawa, and Matusita and Sakka methods, while the Ozawa method was used to determine the Avrami exponent (*n*).

**Figure 7:**
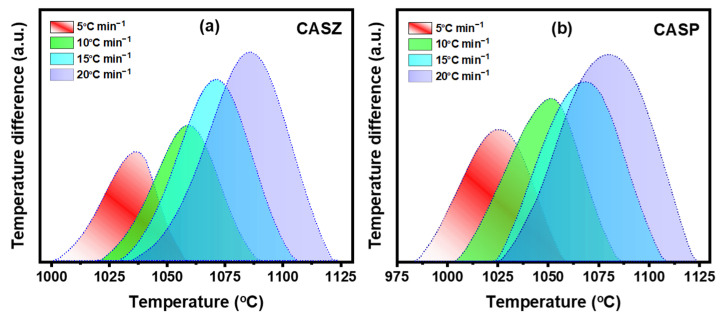
The non-isothermal DTA curves of the glass samples (**a**) CASZ and (**b**) CASP at the crystallization stage at different heating rates (5, 10, 15, and 20 °C min^−1^).

Figure 7 shows the DTA curves of CAS glass samples at 5, 10, 15, and 20 °C min^−1^ heating rates after baseline arrangement. It can be seen from the curves that with the increase in heating rate (*ꞵ*), crystallization peaks shifted towards higher temperature values and the intensity of peaks increased. This situation can be explained by the fact that the amount of the energy requirement per unit of time for nucleation and the subsequent crystal growth process is high at higher heating rates, while sufficient time is available to transfer the heat in glass at lower heating rates. Therefore, higher heating rates postpone the crystallization process to higher temperatures [47,48,49].

The crystallized volume fraction (*ꭓ*) of glass at a certain temperature can be calculated from the DTA curves using the formula given in Equation (3) [50]:

(3) $$\chi =\frac{A_{T}}{A}$$

In this equation, *A* is the total area of the crystallization peak between the temperatures T_s_ (crystallization onset) and T_f_ (where the crystallization process is complete), while *A_T_* represents the area between T_s_ and a certain T temperature.

**Figure 8:**
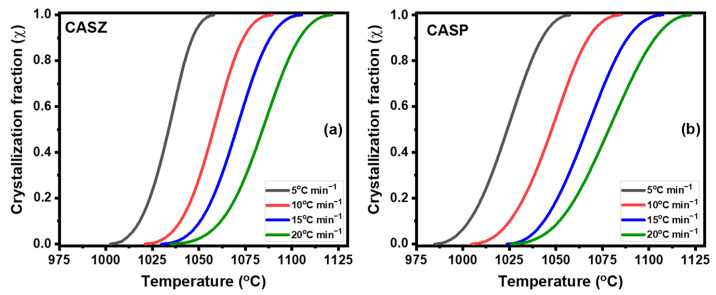
The non-isothermal DTA curves of the glass samples (**a**) CASZ and (**b**) CASP at the crystallization stage, and the crystallization fraction of (**a**) CASZ and (**b**) CASP glass samples at different heating rates (5, 10, 15, and 20 °C min^−1^).

Plots of crystallized fraction (*ꭓ*) as a function of the temperature at the different heating rates of CASZ and CASP glasses exhibit typical sigmoidal curves and are presented in Figure 8. The S-shaped curves describe crystal formation through nucleation and subsequent growth steps within the glass matrix. The above-mentioned crystallization process can be expressed in three stages as it can be distinguished from different characteristic regions (a low slope in the initial stage, an increase in the slope to a buckle point, and finally it approaches the maximum value with the low slope again) in the crystallized fraction (*ꭓ*)–temperature diagrams plotted at different heating rates: (1) a slow beginning period in which nucleation predominates and results in a low conversion rate, (2) a second region with a higher slope in which the nuclei grow rapidly from the glassy matrix, and (3) a saturation stage, where the glassy phase decreases and crystals reach a sufficient size [51,52].

#### 3.4.1. Kissinger Method

**Figure 9:**
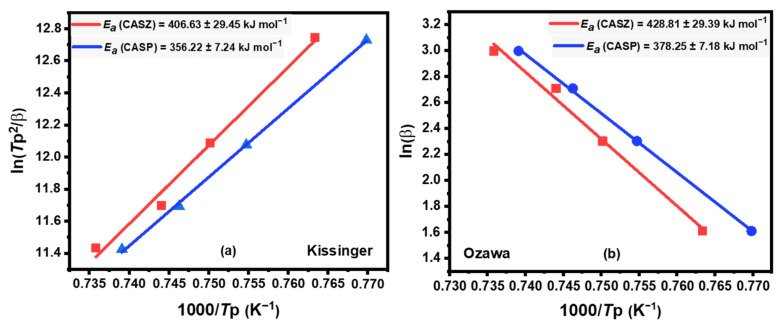
The plots of (**a**) ln *T*_p_^2^/*ꞵ* versus 1000/*T*_p_ of CASZ and (**b**) ln (*ꞵ*) versus 1000/*T*_p_ of CASZ and CASP glasses.

The Kissinger method [53] is a widely employed method for estimating the crystallization activation energy (*E*_a_) in first-order reactions through non-isothermal analysis, based on the variation in the maximum crystallization peak temperatures with the heating rates. The Kissinger equation for determining the crystallization activation energy, *E*_a_, can be expressed according to Equation (4) [54] as below:

(4) $$\ln\left(\frac{{T_{p}}^{2}}{\beta }\right)=\frac{E_{a}}{RT_{p}}+constant$$

where *ꞵ* is the heating rate, *T*_p_ is the temperature at which the crystallization peak is at its maximum, and *R* is the gas constant. The activation energy of crystallization (*E*_a_) can be calculated from the slope of ln (*T*_p_^2^/*ꞵ*) versus 1000/*T*_p_ plots (Figure 9a), which were plotted using the linear fitting method. The calculated crystallization activation energies (*E*_a_) of CASZ and CASP glasses using the Kissinger method are 406.63 ± 29.45 and 356.22 ± 7.24 kJ mol^−1^, respectively.

#### 3.4.2. Ozawa Method

The Ozawa method is another favorable approximate model for the calculation of the crystallization activation energy (*E*_a_) and considers the relationship between the heating rate (*ꞵ*) and crystallization peak temperature (*T*_p_). The activation energy for crystallization (*E*_a_) can be determined by using Equation (5) [55]:

(5) $$\ln\left(\beta \right)=-\frac{E_{a}}{RT_{p}}+constant$$

where *R* is the gas constant. The plots of ln (*ꞵ*) versus 1000/*T*_p_ for CAS glasses are given in Figure 9b and the values of the activation energies (*E*_a_) are calculated from the slope of each line. The Ozawa crystallization activation energies (*E*_a_) of CASZ and CASP glasses were found to be 428.81 ± 29.39 and 378.25 ± 7.18 kJ mol^−1^, respectively.

#### 3.4.3. Matusita Method

The Ozawa and Kissinger approaches, although widely used for kinetic calculations, are thought to have some limitations for estimating the kinetic parameters of amorphous structures such as glasses, since they do not consider the nucleation and crystal growth steps [56]. Therefore, Matusita presented a different approach based on the number of nuclei that varies in the crystal growth stage during the crystallization process, taking into account the growth mechanism (surface or bulk crystallization) and the dimensionality of crystals in glass [57]. Matusita described the activation energy of crystal growth (*E*_a_) as

(6) $$\ln\left[-\ln\left(1-\chi \right)\right]=-n\ln\beta -1.052m\frac{E_{a}}{RT}+constant\;\;\;\;\;\;$$

where *x* is the crystallized volume fraction, *ꞵ* is the heating rate, *T* is the temperature at which the crystallized volume fraction reaches a particular value, *R* is the gas constant, *n* is the Avrami parameter, and *m* is the dimensionality of crystals. In this equation, if the glass contains a sufficient number of nuclei before the heat treatment, *n* is equal to m, but if the nucleation occurs during the heat treatment process, in other words, glass contains no nuclei initially, *n* is equal to *m* + 1 [58].

Among the kinetic parameters, the Avrami constant (*n*) is a parameter that indicates the crystallization mechanism, including volume or surface crystallization along with the dimensionality of crystal growth, as depicted in Table 4, and can be calculated using Equation (7):

(7) $$\ln\left[-\ln\left(1-\chi \right)\right]=-n\ln\beta +constant\;\;\;\;$$

**Figure 10:**
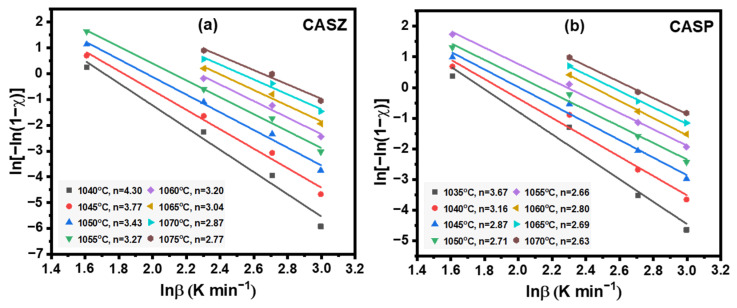
Plots of ln[−ln(1 − *ꭓ*)] versus ln (*ꞵ*) of (**a**) CASZ and (**b**) CASP glasses at selected temperatures for determining Avrami parameters (*n*).

From Equation (7), the value of the Avrami constant (*n*) is calculated from the slope of the ln[−ln(1 − *ꭓ*)] versus ln (*ꞵ*) curves, plotted for different heating rates at eight selected temperatures for CASZ and CASP glasses. The Avrami constant values, calculated as a function of the selected temperature, are shown in Figure 10. From the linear fit curves, it was determined that the *n* values for CASZ glasses ranged from 2.77 to 4.30, while for CASP glass the range was 2.63 to 3.67. The average calculated *n* values for these compositions were 3.33 and 2.89, respectively. An Avrami parameter value between zero and one implies the occurrence of surface crystallization, while a value greater than one signifies the presence of volume crystallization within the structure. Volume crystallization generally occurs in three distinct modes: *n* = 2 denotes one-dimensional crystal growth, *n* = 3 signifies two-dimensional crystal growth, and *n* = 4 points to three-dimensional crystal growth [59]. Although the Avrami constant was calculated to be close to three on average for both glass compositions, the results obtained from the calculations indicate that the *n* index is not constant and decreases inversely with increasing temperature. Specifically, for CASZ glass, the *n* index decreases from 4.30 to 2.77 in the range of 1040–1075 °C, and similarly, for CASP glass, it decreases from 3.67 to 2.63 within the range of 1035–1070 °C. This suggests that the nucleation and crystal growth processes in these glasses do not remain constant throughout the crystallization period. At the beginning of crystallization, three-dimensional crystal growth is dominant in the CAS glasses; however, as crystallization progresses, the growth mechanism tends to shift toward one-dimensional crystal growth. From the perspective of crystal morphology, SEM images of both CAS-coded glass-ceramic samples reveal that the small-sized crystals, more frequently observed at lower sintering temperatures, transform into needle-like and then rod-like forms as the sintering temperature increases. When interpreted in conjunction with the calculations, it can be concluded that crystals initially grow three-dimensionally in the early stages of crystal formation; however, as phase formation progresses, growth becomes one-dimensional with a significant anisotropy, resulting in the formation of needle-like and then rod-like crystals. Karamanov et al. [60] investigated the variation in the Avrami parameter for glass powders with different particle sizes forming diopside using non-isothermal methods and calculated that the Avrami constant varies from 2.54 to 1.35 depending on the method used (Ozawa or Augis–Bennett). The authors reported that three-dimensional particle growth predominates in finer powders and in the early stages of crystallization, while in coarser powders the formation of dendrite-like crystals occurs later with significant anisotropy. A similar phenomenon was also reported by Lu et al. [61] regarding the effect of particle size on the properties of wollastonite glass-ceramics.

In addition, due to the presence of nucleating agents derived from natural raw materials in the glass composition, the Matusita activation energy calculations in this study assumed *m* and *n* to be equal.

**Figure 11:**
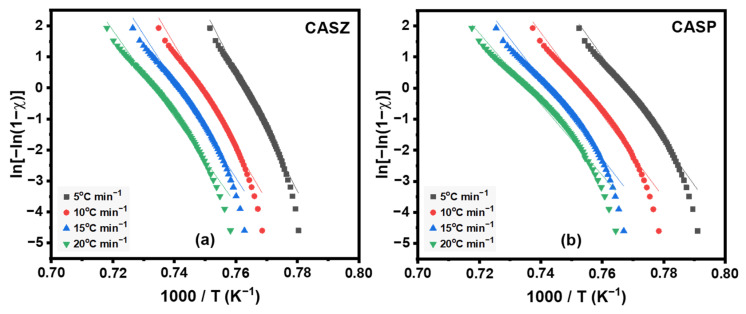
The plots of (**a**) ln *T*_p_^2^/*ꞵ* versus 1000/*T*_p_ of CASZ and (**b**) ln (*ꞵ*) versus 1000/*T*_p_ of CASZ and CASP glasses.

Figure 11 shows ln[−ln(1 − *ꭓ*)] versus 1000/T plots for CAS glasses at different heating rates, where the slope of these curves gives the Matusita crystallization activation energy (*E*_a_). The break in the linearity of the curves for all heating rates at high temperatures is ascribed to the saturation of the nucleation sites during the latter stages of the crystallization process or the limitation of crystal growth due to the small particle size [62,63,64]. From the slopes of the curves, average Matusita *E*_a_ values were calculated as 409.20 ± 7.10 kJ mol^−1^ and 369.44 ± 7.16 kJ mol^−1^ for CASZ and CASP compositions, respectively, and the results are shown in Table 5, compared with values obtained using other methods mentioned above. The table shows that the activation energy values obtained using the Matusita method and those obtained using the Ozawa and Kissinger methods are highly consistent, although there are minor differences depending on the method used.

As is well known, activation energy refers to the energy barrier that is required for the transition from the glassy state to the crystalline form. In this context, it is concluded that the higher the *E*_a_ value, the more energy is required to precipitate the crystals in the glassy matrix [51,59]. The findings of this study indicate that the activation energy required for the crystallization of CASP glass is lower than that in CASZ glass; in other words, the crystallization process of CASP glass can occur more easily and faster.

In the literature, several studies have investigated the crystallization kinetics of CaO-Al_2_O_3_-SiO_2_-based glass-ceramics. For instance, Zhu et al. [65] examined the effect of TiO_2_ content on the crystallization behavior and properties of CAS glass-ceramic fillers, calculating the crystallization activation energy within a range of 460.57 kJ mol^−1^ to 323.59 kJ mol^−1^. They reported that an optimal content of TiO_2_, acting as a nucleating agent, could effectively reduce the crystallization activation energy in this system. In another study, Shi et al. [66] focused on the production and characterization of CAS glass-ceramics with molybdenum tailings and observed that increasing the molybdenum tailing content in the initial composition led to a shift in crystallization peaks to lower temperatures, with the crystallization activation energy decreasing from 276.09 kJ mol^−1^ to 225.81 kJ mol^−1^. This trend was attributed to Fe_3_O_4_ and TiO_2_, present in the molybdenum tailings as network-modifying cations, which disrupt the glass network structure and thus promote crystallization. In the study by Fang et al. [67], which investigates the addition of Li_2_O to CAS glass-ceramics, they reported that the characteristic temperatures decreased with Li_2_O addition, significantly lowering the crystallization activation energy. In our study, the trends in crystallization activation energy values, calculated through various methods and as dependent on composition, are consistent with those reported in other studies on the CAS system and are seen to be strongly influenced by the content of network-modifying oxides.

#### 3.4.4. Local Activation Energy

The activation energy values calculated by the different model-fitting methods represent the average value for the whole crystallization process, but they do not provide any information about the activation energy at specific stages of the crystallization. However, it has been noted by several authors that the activation energy values change in different crystallization fractions due to the changing nucleation and crystallization behavior during the process [50,55]. The variation in the activation energy can be expressed in terms of local activation energy, which can be determined from the non-isothermal DTA results of experiments at different heating rates. In the present study, the local activation energy (*E*_a_) was determined using the Ozawa method according to the following relationship [68]:

(8) $$\ln\left(\beta \right)=-1.0516\;\frac{E_{a}}{RT}+constant$$

where *β* is the heating rate, *E*_a_ is the local activation energy, *R* is the gas constant, and *T* is the temperature corresponding to the crystallization fraction.

**Figure 12:**
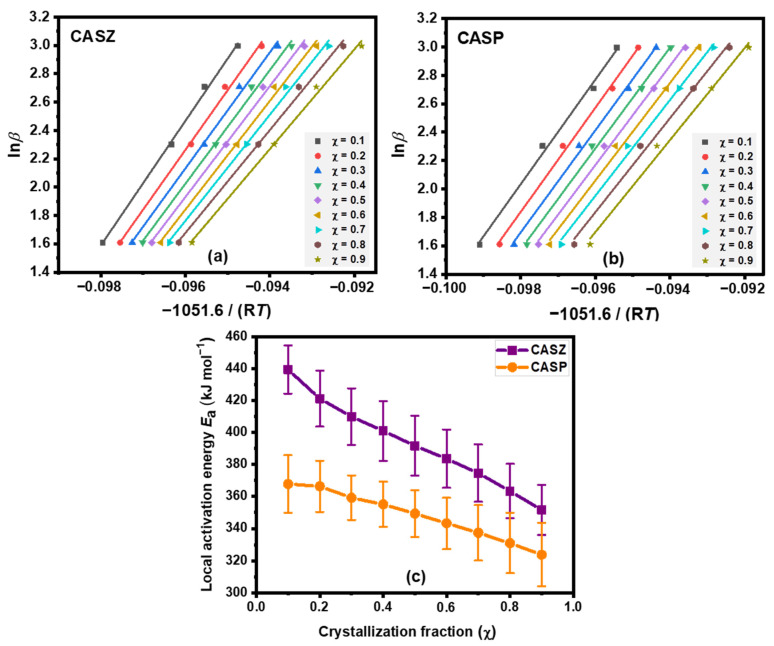
The plots of ln*ꞵ* versus −1051.6/*RT* of (**a**) CASZ and (**b**) CASP glasses at different crystallization fractions and (**c**) the local activation energy (*E*_a_) plot obtained from these curves.

Figure 12a,b show ln *β* versus −1051.6/*RT* plots for CASZ and CASP glass compositions at different crystallization fraction values (*χ* = 0.1–0.9). The local activation energy value *E*_a_ was determined from the slopes of the linear regression lines of these curves, plotted at various heating rates. Figure 12c illustrates the correlation between *E*_a_ and the crystallization fraction (*χ*). As seen, the *E*_a_ values of CASZ and CASP glasses were at their highest level at the early stages of crystallization (*χ* = 0.1), whereas they gradually decreased as the crystallization fraction increased. At the initial stage of crystallization, the nucleation process can be triggered at high local activation energy values due to the increase in crystalline regions with a high diffusion rate in the glassy matrix [58]. After this stage, crystal growth occurs simultaneously with nucleation. In the final period (*χ* = 0.9), the growth of the nuclei reaches saturation, resulting in a lower local activation energy requirement for crystallization compared to the early stage [58,69]. As a result, depending on the crystallization fraction, the *E*_a_ values of CASZ and CASP glasses are approximately in the range of 352–439 kJ mol^−1^ and 324–368 kJ mol^−1^, respectively. The average values of the local activation energy calculated by the Ozawa method in the range of 0.1 ≤ *χ* ≤ 0.9 are 392.96 ± 17.39 kJ mol^−1^ for CASZ and 348.15 ± 16.46 kJ mol^−1^ for CASP. These results are in good agreement with the activation energy values calculated using other model-fitting methods in this study.

### 3.5. FTIR Analysis

**Figure 13:**
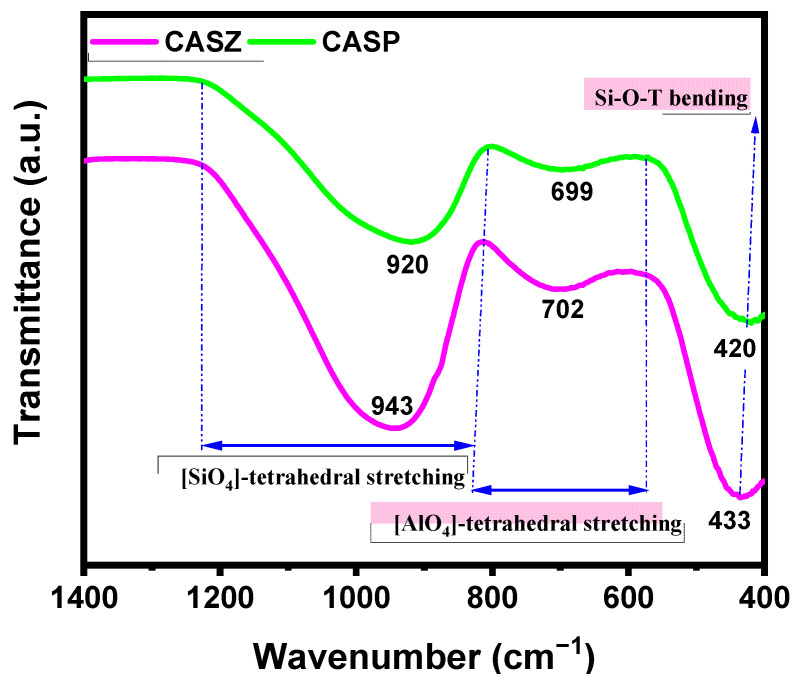
FTIR spectra of CAS glasses.

The tendency of glasses to crystallize, or in other words, the activation energy barrier (*E*_a_) required for the transformation from glass to glass-ceramic, is directly related to the chemical composition of the base glass and therefore to its network structure. In this regard, the FTIR spectra in the range of 1400–400 cm^−1^ of CAS-coded glasses were examined to evaluate the structure of precursor glass powders, as depicted in Figure 13. As can be observed, the spectra in this frequency range can be divided into three main bands, which is typical of alumina silicate glasses. The band within the range of 1200–800 cm^−1^ is attributed to the asymmetric stretching vibration of [SiO_4_] tetrahedra [70], while the band between 800 and 600 cm^−1^ represents the stretching vibrations of [AlO_4_] tetrahedral units [71]. The band located at approximately 600–400 cm^−1^ is due to the bending vibration of bridging oxygen (BO) in Si-O-Al and Si-O-Si linkages [72].

The structure of CaO-Al_2_O_3_-SiO_2_ (CAS) system glasses fundamentally consists of [AlO_4_] and [SiO_4_] tetrahedral units, which serve as glass network formers, interconnected through oxygen atoms sharing a common corner known as bridging oxygen (BO). As is known, since the ionic radii of Al^3+^ and Si^4+^ are close to each other, Al^3+^ can substitute for Si^4+^ in the tetrahedral order. However, this partial substitution due to the presence of Al_2_O_3_ in the glass structure leads to some changes in the network structure of the glass. Since Al^3+^ ions are in fourfold coordination like Si^4+^ ions, a charge deficit occurs in the glass network due to the charge disparity between the ions [73,74]. In the aforementioned system, Ca^2+^ can act as a charge compensator to maintain the electrical charge balance in the structure, while its excess acts as network modifiers that weaken the tetrahedral network by breaking Si-O-Si, Al-O-Al, and Si-O-Al bonds [75,76]. Oxygen ions that bond with cation ions are referred to here as non-bridging oxygens (NBOs). The introduction of more alkaline (M^+^) and alkaline earth (M^2+^) cations into the glass network from the natural raw materials used, as in this study, significantly reduces the degree of polymerization of the glass network.

In the present study, the width and position of the broad absorption bands observed in the 1200–800 cm⁻^1^ range are indicative of the degree of polymerization of the glass network with respect to the relative content of Q^n^ species in the glass structure. Here, n refers to the number of bridging oxygens per tetrahedral unit and can vary between zero and four. The main absorption band tends to shift to lower wave numbers as n decreases [67,76,77]. As shown in Figure 13, the broad band in this range of the CASZ and CASP glasses exhibits a similar trend overall. However, the band of the CASP-coded sample is slightly broader compared to CASZ, and the [SiO_4_] tetrahedral center shifts from higher to lower wavenumbers (CASZ~943, CASP~920). Furthermore, it is observed that the [AlO_4_] tetrahedral stretching (CASZ~702 and CASP~699) and the Si-O-T bending hollows (CASZ~433 and CASP~420) become shallower in CASP compared to CASZ; in other words, the curves are smoothed, indicating that the [AlO_4_] tetrahedral content and the number of Si-O-T complex structures in the glassy network decrease [72,78]. This phenomenon indicates that the higher alkali and alkaline earth metal content in CASP glasses due to the raw materials used reduces the stability of the glass network and promotes depolymerization. The lower degree of polymerization of the network structure for CASP-coded glasses facilitates crystallization, resulting in a lower crystallization peak temperature and activation energy (*E*_a_) for this composition.

### 3.6. Physical Properties of Glass-Ceramics

**Figure 14:**
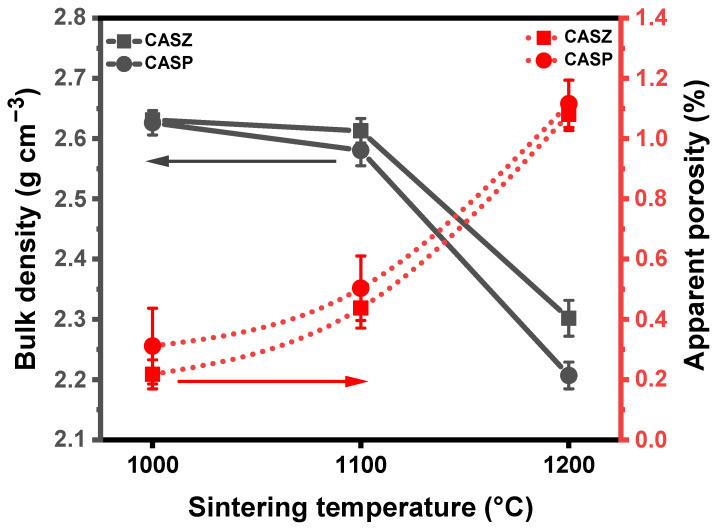
Bulk density and apparent porosity plots of CAS-coded glass-ceramics sintered at different temperatures for 1 h.

Figure 14 presents comparative plots illustrating the bulk density and apparent porosity of CAS glass-ceramic samples sintered for 1 h at various temperatures. The results indicate that increasing sintering temperatures resulted in a gradual decrease in the density of the glass-ceramic samples, accompanied by an increase in apparent porosity. The CASZ glass-ceramic sample sintered at 1000 °C exhibited the highest density value of 2.63 ± 0.06 g cm^−3^, while the highest density among CASP samples, 2.62 ± 0.02 g cm^−3^, was observed under the same sintering condition. As the sintering temperature increased to 1100 °C, the calculated bulk density of CASZ and CASP glass-ceramics was 2.61 ± 0.02 and 2.58 ± 0.02 g cm^−3^, respectively. Following the final sintering at 1200 °C, the density values decreased to 2.30 ± 0.03 g cm^−3^ for CASZ and 2.20 ± 0.02 g cm^−3^ for CASP samples. The evaluation of the apparent porosity results shows that the porosity increases as the sintering temperature increases from 1000 to 1200 °C, which is consistent with the density values measured in CASP- and CASZ-coded glass-ceramic specimens. The apparent porosity percentages for CASZ glass-ceramic samples vary between 0.21 ± 0.04 and 1.08 ± 0.05, while for CASP, they range from 0.31 ± 0.12 to 1.11 ± 0.07.

The microstructures of the glass-ceramic samples confirm the crystallization of anorthite, wollastonite, and pseudowollastonite phases in the glassy matrix, depending on the sintering temperature. At a sintering temperature of 1000 °C, smaller crystals were visible in the microstructure of CASZ, while larger, randomly distributed crystals were found in the microstructure of CASP glass-ceramics. As the sintering temperature increased gradually, the crystal size in both glass-ceramic samples grew continuously, while their number decreased. Concurrently, as the crystal sizes increased, both the size and the number of detected pores within the structures also increased. The density enhancement observed in glass-ceramic materials is attributed to crystallization originating from the glassy matrix. During crystallization, particle rearrangement occurs, while sintering proceeds via a viscous flow mechanism. This is followed by continuous grain growth which allows for further densification. Grain growth occurs at high temperatures due to the decreased viscosity of the matrix material, which enhances its wetting capability and facilitates easier mass transfer [79,80]. This phenomenon also provides an explanation for how elevated sintering temperatures result in the generation of greater quantities of crystalline phases, increased crystal sizes, and enhanced crystallinity percentages, accompanied by a reduction in the amorphous phase content. However, during the transformation from the glassy state to a glass-ceramic, the crystalline phases that precipitate in the glassy matrix and grow due to temperature effects can sometimes negatively impact the achievement of full densification. This effect can be summarized as a reduction in the quantity of the glassy phase due to the consumption of SiO_2_ in the structure during the transformation from glass to glass-ceramic, along with an increase in the viscosity of the glassy phase caused by ion diffusion from the glass to the crystals during this process [81]. The gradual decrease observed in the bulk density values of the glass-ceramic samples in the present study with increasing sintering temperature and, on the contrary, the increase in the apparent porosity values are thought to be due to the fact that the increasing matrix viscosity and the decreasing amount of the glassy phase prevent the compacting of the structures in response to the aforementioned crystalline phases precipitated in the glassy matrix and growing rapidly under the influence of temperature. This phenomenon not only provides an explanation for the observed increase in porosity content in the microstructures of CAS glass-ceramic samples with rising sintering temperatures and correspondingly larger crystal sizes, but it also offers insight into the higher density exhibited by CASZ glass-ceramic samples with smaller, more numerous crystals compared to CASP samples with larger crystals. Similarly, a number of studies in the literature have reported that when crystallization occurs concurrently with sintering, viscous flow is impeded, preventing complete densification and resulting in structures that are fully or partially crystallized, less dense, and more porous [82,83].

### 3.7. Mechanical Properties of CAS Glass-Ceramics

**Figure 15:**
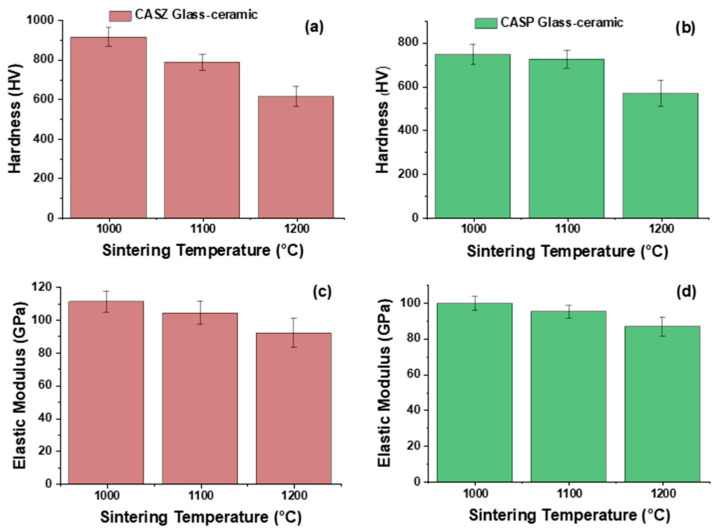
Vickers hardness of (**a**) CASZ and (**b**) CASP and elastic modulus of (**c**) CASZ and (**d**) CASP glass-ceramics at different sintering temperatures.

Figure 15 illustrates a comparative analysis of nano-hardness measurements and elastic modulus values for CASZ and CASP glass-ceramics based on the sintering conditions. For each sample, the nano-hardness measurements were repeated five times. The figures reveal that the CAS-based glass-ceramics attained the highest hardness values at the initial sintering temperature of 1000 °C, with CASZ and CASP glass-ceramics exhibiting Vickers hardness values of 916 ± 46 HV and 747 ± 47 HV, respectively. As the sintering temperature increased, a gradual decrease in the measured nano-hardness values was observed for the CASZ composition. An average hardness value of 787 ± 40 HV was achieved for this composition through sintering processes conducted at 1100 °C, while sintering processes carried out at 1200 °C resulted in a hardness value of 614 ± 50 HV. In a similar manner, glass-ceramic samples coded as CASP exhibited a decreasing trend in hardness values as the sintering temperature increased. The average hardness values achieved for these samples after sintering at 1100 °C and 1200 °C were 726 ± 40 HV and 569 ± 60 HV, respectively. Moreover, the elastic modulus values demonstrate a similar trend to the hardness results, varying between 111 ± 6.3 GPa and 92 ± 9.1 GPa for CASZ glass-ceramics, and between 99 ± 3.9 GPa and 87 ± 5.3 GPa for CASP-coded glass-ceramics, depending on the temperature. The obtained results indicate a decrease of 33% and 24% in hardness values, and 17% and 13% in elastic modulus values, respectively, with increasing sintering temperatures from 1000 °C to 1200 °C for CASZ and CASP-coded glass-ceramics. To achieve high mechanical properties in glass-ceramic materials, it is necessary to develop homogeneous microstructures with appropriate crystal sizes and minimal porosity within the samples. In the current study, it is evident that the hardness and elastic modulus values of glass-ceramics are notably influenced by the emergence and enlargement of porosities within the structure as sintering temperatures increase. It is also worth noting that the increase in the porosity of glass-ceramics as a result of sintering temperatures has a more noticeable effect on the hardness values than the elastic modulus. Similar findings have been reported in the research conducted by Chen et al. [84].

**Figure 16:**
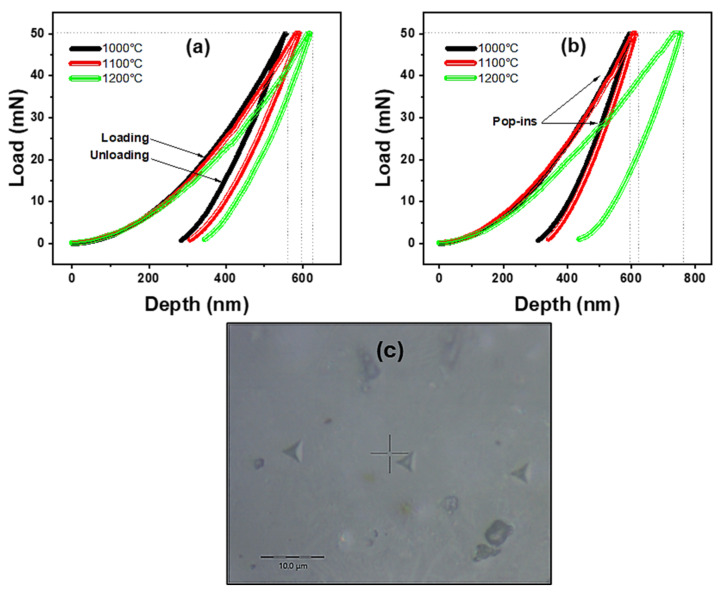
Load–depth curves of (**a**) CASZ and (**b**) CASP glass-ceramics and (**c**) nano-hardness image of CASZ glass-ceramic sintered at 1000 °C.

Figure 16 presents representative load–displacement curves obtained from the indentation tests of CAS-coded glass-ceramics sintered at different temperatures. The maximum load is 50 mN, with maximum penetration depths of 557, 592, and 620 nm for CASZ samples and 597, 614, and 757 nm for CASP at sintering temperatures of 1000, 1100, and 1200 °C, respectively. The larger indentation depths observed as a result of increasing sintering temperatures are also indicative of lower elastic recovery in these samples [84]. Multiple ‘pop-in’ events are discernible in most of the load–displacement curves, except for the CASZ sample sintered at 1000 °C for 1 h. The lengths of these pop-ins increase proportionally with the sintering temperature. It is postulated that the absence of a discernible change in displacement in the aforementioned sample is due to the fact that the potential pop-in lengths are too small to be distinguished. Pop-in phenomena are frequently observed in load–displacement curves plotted during nano-indentation tests and are defined as a sudden increase in indenter displacement under the applied normal load, which is observed as a horizontal plateau in the curves [85,86]. In the present study, the observed pop-in phenomena in CAS-coded specimens’ load–displacement curves are due to increasing porosities during the glass to glass-ceramic transformation, which grow in both number and size with rising sintering temperatures. A high porosity concentration can facilitate the interaction between porosities and pore collapse due to stress concentration, which can lead to micro-crack formation and propagation during indentation, characterized by a sudden displacement, i.e., pop-in, in the load–displacement curves [87]. Considering the density results, it can be interpreted that the pop-in formations observed in the curves increase with the porosity fraction, and at the same time, the length of the plateaus in these curves also increases in direct proportion to the porosity fraction. This correlation is also consistent with the nano-hardness and elastic modulus, which decrease with increasing porosity.

## 4. Conclusions

In this study, CaO-Al_2_O_3_-SiO_2_ (CAS) glass-ceramics were prepared from pumice, zeolite, and eggshell waste as the main raw materials. The higher content of network-modifying metal oxides in the CASP composition produced from pumice, compared to CASZ, where zeolite was used, had a significant effect on the crystallization peak temperature and crystallization activation energy, as well as the mechanical, structural, and physical properties of the glass-ceramics.

- The crystallization peak temperature and crystallization activation energy decreased with the increasing content of modifying metal oxides in the glass structure. While the activation energy required for the crystallization of CASZ, calculated using different approaches, ranged between 406 and 428 kJ mol^−1^, it ranged between 356 and 378 kJ mol^−1^ for CASP. The average local activation energy values calculated from different crystallization fractions of the CASZ and CASP glasses were 392.96 ± 17.39 kJ mol⁻^1^ and 348.15 ± 16.46 kJ mol⁻^1^, respectively.
- The Avrami parameter (*n*) for CASZ and CASP glasses was calculated as 3.33 and 2.89 on average from the determined temperatures, respectively. However, the value of *n* is not constant and tends to decrease with increasing temperature. This indicates that the crystal growth in CAS glasses varies from three-dimensional to one-dimensional during crystallization and the crystallization mechanism is volume crystallization.
- XRD analyses revealed that anorthite and wollastonite were the crystalline phases detected in both glass-ceramics after sintering at 1000 °C and 1100 °C, while increasing the temperature to 1200 °C resulted in the disappearance of wollastonite peaks and the formation of intense peaks corresponding to the pseudowollastonite crystalline phase.
- SEM examinations demonstrated the existence of porosity within the glass-ceramic samples. Initially, the crystals exhibited a needle-like structure; however, as the sintering temperature elevated, they underwent morphological changes and exhibited rod-like characteristics after the final sintering at 1200 °C.
- The highest bulk density of the glass-ceramic samples was achieved at the initial sintering temperature of 1000 °C, with calculated values of 2.63 g cm^−3^ for CASZ and 2.62 g cm^−3^ for CASP.
- Depending on the sintering temperature, the Vickers hardness of the CASZ-coded glass-ceramics ranged from 916 to 614 HV, while CASP ranged from 747 to 569 HV. Additionally, the elastic modulus was found to vary between 111 and 92 GPa and 99 and 87 GPa for these two compositions, respectively. Increasing sintering temperatures resulted in a decrease in mechanical properties for both glass-ceramics.

## Figures and Tables

**Table 1:** Chemical composition of eggshell, zeolite, and pumice (wt.%).

Compound (wt.%)	Eggshell	Zeolite	Pumice
CaO	51.76	2.0	3.25
Al_2_O_3_	0.04	13.2	13.21
SiO_2_	0.10	71.9	72.45
K_2_O	0.08	3.5	5.07
Na_2_O	0.11	0.3	3.17
Fe_2_O_3_	0.12	1.4	1.94
MgO	0.36	1.1	0.65
TiO_2_	-	0.1	0.14
P_2_O_5_	0.17	-	-
MnO	-	0.1	0.08
SO_3_	0.597	-	-

**Table 2:** Composition of the CAS glass-ceramics (wt.%).

Sample Name (wt.%)	CaO	Al_2_O_3_	SiO_2_
CASZ	27.5	15	57.5
CASP	27.5	15	57.5

**Table 3:** Glass transition (T_g_), crystallization peak (T_p_), and melting (T_m_) temperatures of CAS glasses at different heating rates (5, 10, 15, and 20 °C min^−1^).

Glass	*β* (°C min^−1^)	T_g_ (°C)	T_p_ (°C)	T_m_ (°C)
CASZ	5	734	1037	1241
	10	745	1060	1241
	15	751	1071	1240
	20	759	1086	1240
CASP	5	703	1026	1204
	10	715	1052	1207
	15	721	1067	1204
	20	728	1080	1205

**Table 4:** Avrami parameter (*n*) according to the crystallization mechanism [47].

Crystallization Mechanism		*n*
Volume crystallization	Three-dimensional crystal growth	4
	Two-dimensional crystal growth	3
	One-dimensional crystal growth	2
Surface crystallization		1

**Table 5:** Average activation energy values (*E*_a_) calculated from various kinetic models and the Avrami constant (*n*).

Sample	Kissinger (kJ mol^−1^)	Ozawa (kJ mol^−1^)	Matusita (kJ mol^−1^)	Avrami Constant (*n*)
CASZ	406.63	428.81	409.20	3.33
CASP	356.22	378.25	369.44	2.89

## Data Availability

The original contributions presented in the study are included in the article; further inquiries can be directed to the corresponding author.
